# Cumulative Risk for Periprosthetic Fracture and Operative Treatment Options After Revision Total Hip Arthroplasty with a Modular and Tapered Revision Device—A Consecutive Series of 117 Cases in a Mid-Term Duration

**DOI:** 10.3390/jcm14155321

**Published:** 2025-07-28

**Authors:** Oliver E. Bischel, Matthias K. Jung, Max Pilgrim, Arnold J. Höppchen, Paul M. Böhm, Jörn B. Seeger

**Affiliations:** 1 BG Trauma Center, University of Heidelberg, Ludwig-Guttmann-Str. 13, 67 071 Ludwigshafen, Germany; matthias.jung@bgu-ludwigshafen.de (M.K.J.); max.pilgrim@bgu-ludwigshafen.de (M.P.); 2 Medical Faculty Mannheim, University of Heidelberg, Ludolf-Krehl-Str. 13-17, 68 167 Mannheim, Germany; arnold.hoeppchen@medma.uni-heidelberg.de; 3 Neckar-Odenwald-Hospital Mosbach, Academic Teaching Hospital of Heidelberg University, Knopfweg 1, 74 821 Mosbach, Germany; 4 General Orthopedics, Pläntschweg 25, 81 247 Munich, Germany; info@ortho-boehm.de; 5 Parc Clinic, Am Kaiserberg 2-4, 61 231 Bad Nauheim, Germany; joernseeger@gmx.net

**Keywords:** modular revision stem, tapered revision stem, revision THA, periprosthetic fracture, survivorship analysis

## Abstract

**Background:** Implantation of modularly built-up stems with a tapered and fluted design is currently state of the art in revision total hip arthroplasty (RTHA). Nevertheless, implant-specific major complications like breakage of taper junctions as well as periprosthetic fractures (PPFs) may lead to failure of reconstruction during follow-up. **Methods:** A cohort of 117 cases receiving femoral RTHA by a modular stem was investigated retrospectively with a mean follow-up of 5.7 (0.5–13.7) years. Cumulative risk and potential factors affecting the occurrence of PPFs were calculated with the Kaplan–Meier method. In addition, cases were presented to discuss operative treatment options. **Results:** A cumulative risk of PPF of 12.1% (95% CI: 0–24.6%) was calculated at 13.7 years. Female patients had significantly higher risk compared to male patients (0% after 13.5 years for male patients vs. 20.8% (95% CI: 0.5–41.2%) after 13.7 years for female patients; log-rank *p* = 0.0438) as all five patients sustaining a PPF during follow-up were women. Four fractures were treated by open reduction and internal fixation. Non-union and collapse of the fracture occurred in one patient after closed reduction and internal fixation. **Conclusions:** Postoperative PPF after femoral revision with a modular stem has shown to be a frequent complication within this mid-term follow-up. Female patients were at a significantly higher risk in this aged cohort, indicating osteoporosis as a risk factor. The surgical treatment of PPF with an integrated long-stemmed prosthesis is challenging and thorough considerations of adequate operative treatment of PPFs are strongly advised in order to limit complication rates.

## 1. Introduction

The number of patients presenting with aseptic loosening, infection or mechanical failure has risen steadily during the last decades due to demographic change and an increasing indication for primary total hip arthroplasty (PTHA) in industrial countries. The quantity of cemented revision total hip arthroplasty (RTHA) has declined due to limited fixation as cancellous bone and surface for cement penetration is missing in the defect area of the former implant bed [1]. Impaction bone grafting with cemented stems as one additional development for RTHA can be performed with a good functional outcome and implant durability, especially in moderate and uncontained bony defects [2].

Nevertheless, cementless fixation by bridging the defect region of the proximal femur has become accepted during the last four decades and tapered fluted stems showed favorable results compared to cylindrical devices even in the presence of massive femoral bone loss [3,4]. Modularly built-up devices have been developed to facilitate implantation and adaptation of the implant to the patients’ anatomy or intraoperative findings. Currently, modular systems with tapered and fluted implants are most frequently used for femoral RTHA with reliable mid-term results [5,6], but implant-related major complications leading to failure like the breakage of tapered junctions and its risk factors have been published [7,8,9]. Other complications like the occurrence of a PPF following implantation of a bony integrated modular revision device may also increase during follow-up and may be underestimated as impactful data is rare. According to the annual report of the Swedish Hip Arthroplasty Register of 2020, a re-revision rate of about 5% has to be expected due to a PPF after RTHA [10]. Over one-third of the surgically treated PPFs after PTHA are revised using longer, most often modularly built-up revision devices with distal fixation in order to bridge the fracture [1]. Although similar principles are applicable, operative treatment of bony integrated and long-stemmed prosthesis with a round design filling the tube is demanding and unequally more complex compared to PTHA. Consecutively, restoration of function for hip and knee may be sometimes limited independent of further accompanying factors like bone quality and/or age.

There is less data available that has focused on postoperative PPF after femoral RTHA with a modular revision device and a medium-term follow-up duration. The cumulative risk of PPF was calculated and potential risk factors investigated based on a retrospective analysis. In addition, operative treatment principles to revise a long-stemmed revision prosthesis after PPF are discussed.

## 2. Patients and Methods

### 2.1. Inclusion Criteria and Methods

A retrospective analysis of a consecutive group receiving femoral RTHA using the modular MRP^®^ revision prostheses (Peter Brehm, Weissendorf, Germany) was performed. Table 1 shows basic personal data of the patients and index operation. Only patients with bony integrated stems were included for analysis. Cases with periprosthetic joint infection (PJI) were excluded from the study as (low-grade) infection may prevent bony integration and therefore secondary stability. All cases with aseptic loosening and/or subsidence without secondary stabilization and/or instability were also excluded from the study. One patient was from abroad and was lost to follow-up after 33 days. The patient was not included as no further data was available. A PPF was not determined until the latest follow-up and/or revision of the excluded patients.

Secondary stability due to an at least partial ingrowth of the host bone into the prosthesis was supposed at a minimum follow-up of five weeks. Consecutively, only patients with evidence of a stable implant were integrated into the study to calculate the risk of postoperative PPF.

Appearing PPFs were graded according to the Vancouver classification system [11]. Factors with potential influence on the occurrence of PPFs during follow-up were statistically analyzed. Patient-specific factors were investigated including gender and BMI. In addition, X-ray examination was performed to assess underlying bone defects according to Paprosky et al. [12]. Furthermore, the reconstruction length or diameter as an implant-related influence on the occurrence of a PPF during follow-up was also investigated.

Statistical analysis was performed with JMP 10 for Mac (SAS Institute Inc., Cary, NC, USA). A time-to-event analysis was performed using the Kaplan–Meier method with postoperative PPF as the failure criterion. A 95% confidence interval was given to all survivorship data; the *p*-value for comparing survival curves was calculated with the log-rank-test. A *p*-value ≤ 0.05 was considered significant.

### 2.2. Surgical Technique at Index Operation and Aftercare

Indications leading to the index surgery and further relevant data like age at surgery, gender, underlying preoperative defect or use of bone transplant describing the point of departure at femoral RHTA are listed in Table 1.

The modular device is available with a straight distal anchoring piece in two lengths. The 140 mm option was used in 37 cases and the 200 mm in 3 cases. Preparation of the implant bed of the straight variants was performed with a corresponding taper reamer of the modular system. The 200 mm variant and all longer options are curved (200 mm, n = 67; 260 mm, n = 9; 320 mm, n = 1) and flexible reamers were used for preparation of the femoral canal.

Two days after surgery, patients started with physiotherapy and mobilization. Postoperatively, hip motion was restricted to 60 degrees and partial weight-bearing of 20 kg bodyweight was allowed. X-ray control was scheduled after six weeks and a stepwise increase in weight-bearing with 10–20 kg per week was allowed in case of an inconspicuous result. Aftercare in a wheelchair was performed for at least six weeks in patients not able to safely perform partial weight-bearing. Weight-bearing with half-body weight was affiliated and stepwise increased afterwards with 10–20 kg per week. All other patients received aftercare at specialized rehabilitation units after reaching full weight-bearing.

## 3. Results

### 3.1. Basic Data and Survivorship Analysis

One hundred seventeen patients met the inclusion criteria but eleven patients died during follow-up. Data of the deceased were enclosed until the latest follow-up. Mean time duration until death, mean follow-up, BMI, dimensioning of the used implant and the calculated survivorship with PPF as the endpoint are presented in Table 2.

### 3.2. Periprosthetic Fractures

**Figure 1:**
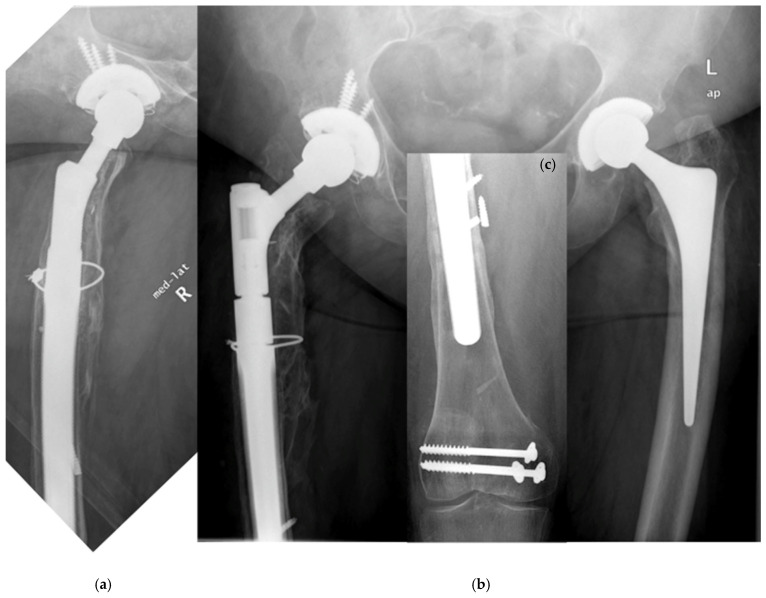
(**a**–**c**) Postoperative situation after femoral RTHA. A former supracondylar fracture was fixed by plate and screws.

**Figure 2:**
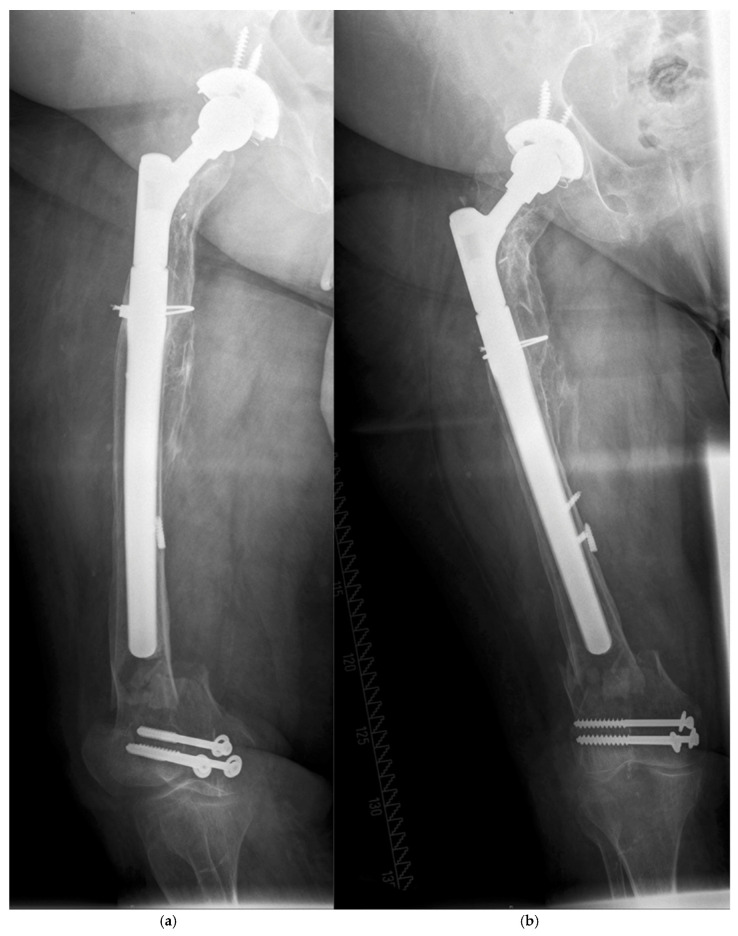
(**a**,**b**) Vancouver type C fracture in the supracondylar area.

**Figure 3:**
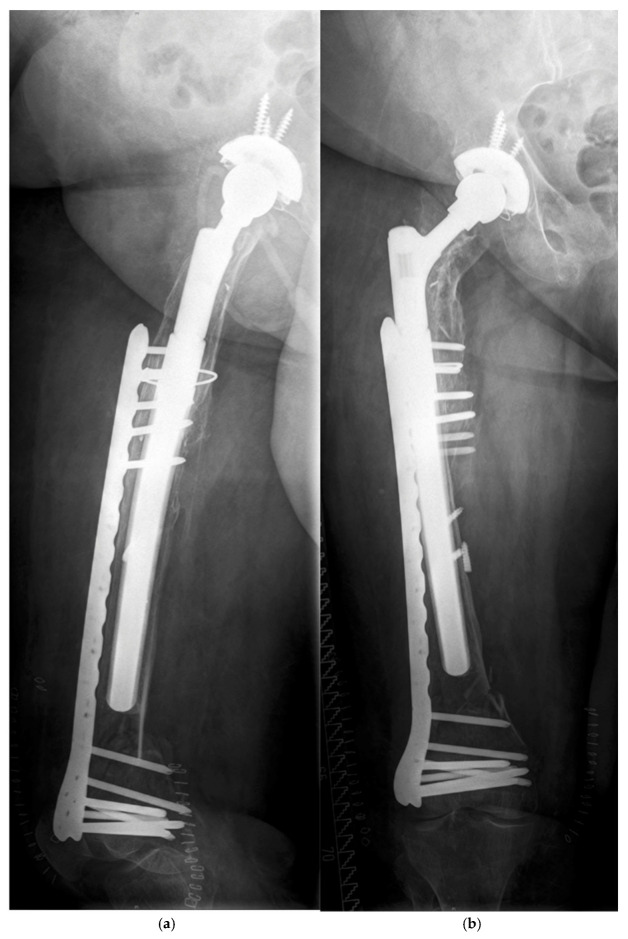
(**a**,**b**) Closed reduction and internal fixation by plate was performed.

**Figure 4:**
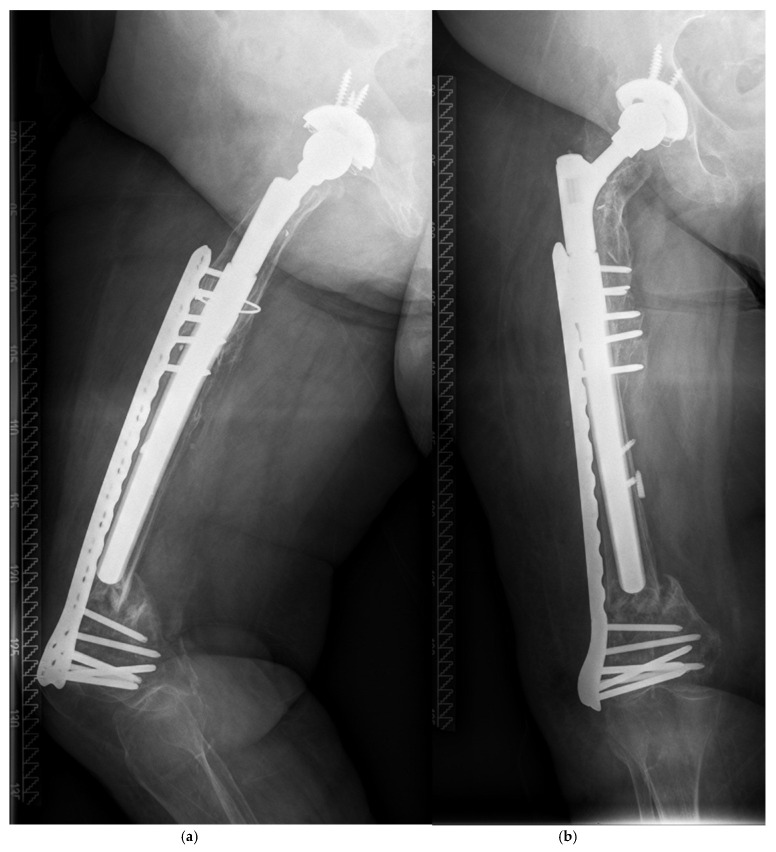
(**a**,**b**) Collapse of the fracture occurred within two months after osteosynthesis.

There were five patients with a PPF during follow-up (Table 3). All fractures occurred after a low-impact trauma event, like stumbling at home, at a mean time of 4.7 (0.1–9.8) years postoperatively (Table 2). All patients showed a displaced fracture near the tip of the prosthesis and were treated surgically. The Vancouver system was used to classify the fractures (Table 3) [11]. Open reduction and internal fixation by plate (ORIF) was performed in four patients. All these fractures after ORIF healed and were stable during further follow-up. Non-union of the fracture and collapse occurred in one patient after closed reduction and internal fixation (CRIF) by plate (Figure 1a–c, Figure 2a,b, Figure 3a,b and Figure 4a,b; Table 3).

### 3.3. Survivorship Analysis and Predictive Risk Factors for PPF

**Figure 5:**
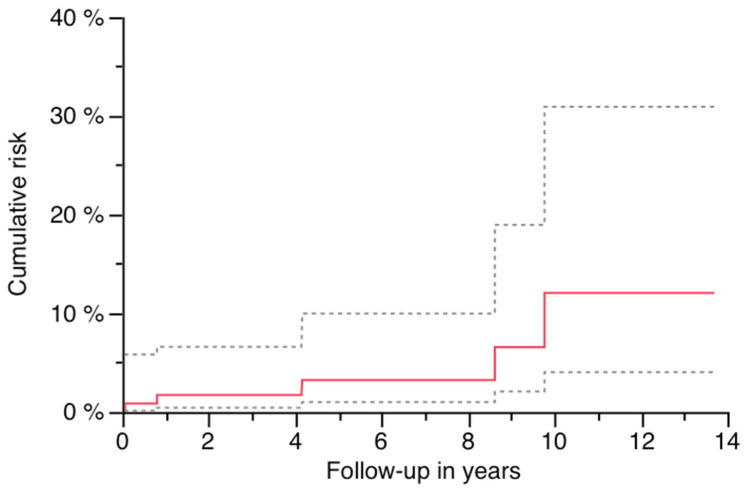
Risk of PPF with 95% CI (dashed lines).

**Figure 6:**
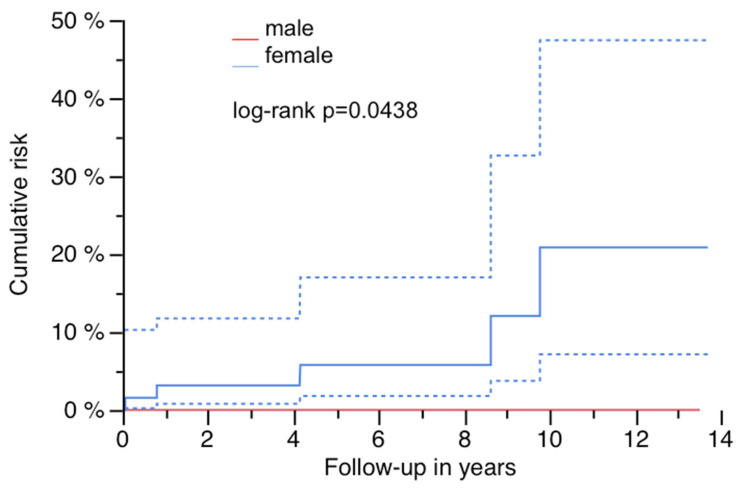
Risk of PPF and gender with 95% CI (dashed lines).

Cumulative risk was 12.1% (95% CI: 0–24.6) at 13.7 years (Figure 5, Table 2). All patients presenting with a PPF were female, revealing gender as a significant factor for the occurrence of a PPF during follow-up (Figure 6; log-rank *p* = 0.0438; Table 2 and Table 3). A significant influence of stem length on PPF could not be found (Table 2), although all PPFs occurred in stems as long or longer than the used median length (250 mm). Preoperative defect situations, diabetes and the diameter of the implant did not affect the risk of PPF during follow-up.

## 4. Discussion

### 4.1. Cumulative Risk of PPF and Predicting Factors

The cumulative risk of PPF with this modular implant was 12% after 14 years and within the overall failure rates due to any cause of this stem in comparable studies and was lower compared to the risk of postoperative PPF after cementless PTHA [5,6,13,14]. This finding is different from other publications including systematic reviews and meta-analysis, which revealed a three times higher odds ratio of sustaining a postoperative PPF after RTHA compared to primary THA [15]. Female gender was associated with a significantly higher risk of PPF during follow-up in this cohort. Osteoporosis was not specifically verified by bone density measurements in this study, but sex and additional factors like high age of patients receiving RTHA may potentiate and may not only be influencing risk factors for PPF after primary THA [16,17,18,19].

### 4.2. Technical Considerations

Although a significant impact between the occurrence of a PPF and used reconstruction length or underlying defect could not be found, fractures occurred without exception in longer curved devices (Table 2). A clear explanation cannot be given to that finding, but longer implants than needed should be avoided as they may even hinder adequate osteosynthesis in the case of potential implant-preserving treatment for PPF.

According to the Vancouver classification, all fractures were of type B1 near the tip or type C, so ORIF or CRIF was performed, respectively. There was one failure of the osteosynthesis with presence of a non-union. This was the only minimally invasive osteosynthesis of the whole cohort (Figure 1a–c, Figure 2a,b and Figure 3a,b). Improper reduction and lack of medial support due to bone defects in the supracondylar region accompanied by systemic osteoporosis led to a collapse of the reconstruction within two months (Figure 4a,b).

This case impressively demonstrates some potential pitfalls of operative treatment of PPF after femoral RTHA. Osteosynthesis around an ingrown and stable long-stemmed prosthesis with a round diameter filling the whole bone canal is somewhat challenging. In contrast to cylindric stems and the option of a strictly perpendicular positioning of the plate to the stem, bicortical fixation of screws is most often not possible, especially near the isthmus even with the high quality of current surgical materials. Suitable fixation with the use of attachment plates specifically developed for the treatment of PPFs is also limited.

Especially in supracondylar fractures, the use of an additional medial plate or cortical strut graft fixed with cerclages and/or morsellized allograft bone to improve local biomechanical conditions should be considered [20,21]. Compared to single lateral plating, bilateral bridging with straight or helically formed plates is biomechanically superior and may prevent breaking out of the screws, especially in cases with a deficient cortical frame and poor bone quality due to osteoporosis [22,23].

Additional cable fixation of the plate proximally in the case of monocortical fixation [24] and near the isthmus with or without adaption of cortical strut grafts may be an option to bridge the forces in a physiological way and to secure fracture healing. In our opinion, open reduction may be often necessary for better assessment of the local situation and to address the above-mentioned pitfalls.

Although we did not see any fracture of type B2 or B3 in this series, the following aspects might be helpful in the case of patients presenting with PPFs of these types after RTHA. If primary stability can be provided without subsidence within the first weeks postoperatively, secondary stability due to bony ingrowth will be safely achieved, resulting in a reported low amount of failures due to aseptic loosening [5,6]. In our experience and according to the already mentioned literature, most cases with aseptic (early) failure of this stem or similar devices with the same tapered and fluted ‘Wagner’ design are related to technical mistakes like the implantation of an under-dimensioned stem or intraoperative PPF. Moreover, infection should be thoroughly excluded.

Adverse results have been reported in review articles after treatment of type B2 or B3 fractures either with ORIF or RTHA. Similar revision rates and good functional and clinical results in selected patients with low functional demand and high anesthetic risks due to multiple comorbidities may profit from ORIF as the operative effort is lower [25,26]. In contrast, higher revision rates have been reported in another review article after ORIF, especially of type B2 but also of type B3 fractures [27]. Nevertheless, ‘de-escalation’ may be one option to treat PPF with a loose or under-dimensioned but long implant and a fracture located more distally. Presupposition is a short but intact area of the proximal femur, enabling enough primary stability to implant a (much) shorter device. If endofemoral exposure may be too time-consuming or at high risk of uncontrolled additional bone damage, transfemoral removal with a short osteotomy of the proximal region may also be an option. Biomechanically, ORIF can be performed more safely after ‘de-escalation’ and in a more physiological way as a longer distance of native bone is not filled with an implanted prosthesis. Using a tapered and fluted revision device like the MRP with the above-mentioned techniques. 

### 4.3. Limitations

This is a monocentric investigation with a retrospective study design. Although it is a consecutive series of operated patients using the same implant, there may be bias due to the study protocol, a missing control group and due to the fact that it is only a mid-term follow-up period. On the other hand, the cohort consists of an adequate number of patients. In addition, a Kaplan–Meier analysis may be sufficient to calculate a time-dependent risk for the occurrence of a PPF after femoral RTHA with this implant. As similar investigations are missing so far, comparison of the results and proof of methodological errors are limited.

## 5. Conclusions

Gender is an influencing factor for osteoporosis and predictive for the occurrence of a PPF during follow-up after cementless femoral RTHA using this modular tapered and fluted revision device. Overall, the cumulative risk of PPF was relatively high in this mid- to long-term follow-up. Thorough considerations are strongly advised for the sufficient operative treatment of PPFs.

## Figures and Tables

**Table 1:** Basic data of the patients and index operation.

	MRP Brehm
Original cohort:	130
Excluded patients due to the following:	
Infection	7
Subsidence within 6 weeks po./and instability	4/2
Aseptic loosening	1
Lost to follow-up	1
Included no. of patients	117
Implanted between (year)	2003–2009
Indication:	
Aseptic loosening	51 (43.6%)
Septic two-stage revision	50 (42.7%)
Periprosthetic fracture	15 (12.8%)
Instability	1 (0.9%)
Surgeons involved	8
Sex (w/m)	64 (54.7%)/53 (45.3%)
Operated side (r/l)	57 (48.7%)/60 (51.3%)
Mean age at surgery (range) in years	67.87 (36.87–85.37)
Surgical approach:	
Transfemoral	17 (14.5%)
Hardinge/transgluteal	100 (85.5%)
Preoperative bone defect (Paprosky)	
Grade 1	18 (15.4)
Grade 2	73 (62.4%)
Grade 3A	2 (1.7%)
Grade 3B	18 (15.4%)
Grade 4	6 (5.1%)
Bone transplant at the femur:	
Total	19 (16.2%)
Autogeneous	3 (2.6%)
Allogeneous	16 (13.7%)
Morsellized	14 (12.0%)
Bulk/strut graft	3 (2.6%)
Both morsellized and bulk graft	2 (1.7%)

**Table 2:** Results.

	MRP Brehm
Mean follow-up in years *	5.7 (0.5–13.7)
Death during follow-up	n = 11 (9.4%)
Follow-up of deceased patients in years	4.1 (0.5–13.7)
Mean reconstruction length (range)/median in mm	249.5 (190–370)/250
Mean stem diameter (range)/median in mm	17.0 (13–30)/17
BMI in kg/m^2^	27.8 (16.3–47.7)
Overweight	74 (63.2%)
Normal weight	41 (35.0%)
Underweight	2 (1.7%)
PPF during follow-up	5 (4.3%)
Follow-up until PPF (ys.)	4.7 (0.1–9.8)
Overall risk (95% CI) of PPF after years in %	12.1 (0–24.6) after 13.7 ys.
Risk of PPF and sex: female vs. male after years in %	20.8 (0.5–41.2) after 13.7 ys.
	vs.
	0 after 13.5 ys.
	Log-rank *p* = 0.0438
Risk of PPF and stem length: shorter devices vs. ≥ median length after years in %	0 after 11.2 ys.
	vs.
	16.7 (0–33.7) after 13.7 ys.
	Log-rank *p* = 0.1597

* Failures due to periprosthetic fracture included.

**Table 3:** Periprosthetic fractures.

Patient, Age at Surgery (ys.), Gender	Indication ^#^	Bone Defect Paprosky et al.	Approach ^§^	BMI (kg/m^2^)	Diabetes (y/n)	PPF Postop. (ys.)	Vancouver Classifi-cation	Conservative (C) vs. Operative Therapy (O)	Stem/Recon-Struction Length/Diameter (mm) *
77, f.	TSR	4	TG	20.8	y	4.2	C	O (ORIF by Plate)	290/14
68, f.	AL	2B	TG	22.5	n	0.8	C	O (ORIF by Plate, Liss ^©^)	250/18
73 ^+^, f.	AL	3B	TG	34.4	n	8.6	C	O (MIS by Plate, VA ^©^)	290/21
75, f.	AL	2B	TG	25.9	n	9.8	C	O (ORIF by Plate, VA ^©^)	250/19
76, f.	AL	2A	TG	31.8	n	0.1	B1	O (ORIF by Plate, Liss ^©^)	240/22

^#^ Two-stage revision (TSR); aseptic loosening (AL). ^§^ Transgluteal-lateral/Hardinge (TG). * Stem length of the MRP system: distal anchoring device and neck; all stems were curved. ^+^ Closed reduction and minimally invasive osteosynthesis. This patient had a former supracondylar fracture many years before RTHA. Collapse of the fracture with non-union occurred within two months (Figure 1a,b and Figure 4a,b).

## Data Availability

The datasets presented in this article are not readily available because the data are part of an ongoing study. Requests to access the datasets should be directed to the corresponding author.
